# A systematic review on reporting quality of economic evaluations for negotiated glucose-lowering drugs in China national reimbursement drug list

**DOI:** 10.1186/s12913-024-11001-3

**Published:** 2024-05-01

**Authors:** Shi-Yi Bao, Liu Liu, Fu-Ming Li, Yi Yang, Yan Wei, Hui Shao, Jian Ming, Jun-Tao Yan, Ying-Yao Chen

**Affiliations:** 1 NHC Key Laboratory of Health Technology Assessment, School of Public Health, Fudan University, Shanghai, 200032 People’s Republic of China; 2 Centers for Disease Control and Prevention, University of Florida, Gainesville, FL 32610-0496 USA

**Keywords:** Quality evaluation, Economic evaluations, National Reimbursement Drug List (NRDL), Systematic review, Glucose-lowering drugs

## Abstract

**Background:**

This study aimed to examine the reporting quality of existing economic evaluations for negotiated glucose-lowering drugs (GLDs) included in China National Reimbursement Drug List (NRDL) using the Consolidated Health Economic Evaluation Reporting Standards 2013 (CHEERS 2013).

**Methods:**

We performed a systematic literature research through 7 databases to identify published economic evaluations for GLDs included in the China NRDL up to March 2021. Reporting quality of identified studies was assessed by two independent reviewers based on the CHEERS checklist. The Kruskal–Wallis test and Mann–Whitney U test were performed to examine the association between reporting quality and characteristics of the identified studies.

**Results:**

We have identified 24 studies, which evaluated six GLDs types. The average score rate of the included studies was 77.41% (SD:13.23%, Range 47.62%-91.67%). Among all the required reporting items, characterizing heterogeneity (score rate = 4.17%) was the least satisfied item. Among six parts of CHEERS, results part scored least at 0.55 (score rate = 54.79%) because of the incompleteness of characterizing uncertainty. Results from the Kruskal–Wallis test and Mann–Whitney U test showed that model choice, journal type, type of economic evaluations, and study perspective were associated with the reporting quality of the studies.

**Conclusions:**

There remains room to improve the reporting quality of economic evaluations for GLDs in NRDL. Checklists such as CHEERS should be widely used to improve the reporting quality of economic researches in China.

**Supplementary Information:**

The online version contains supplementary material available at 10.1186/s12913-024-11001-3.

## Introduction

Diabetes imposes substantial economic and health burdens on individuals and society. According to International Diabetes Federation Diabetes Atlas, 451 million adults were estimated to be suffering from diabetes, and global diabetes-related health expenditures were estimated at 966 billion US dollars (USD) in 2021, projected to reach 1,054 billion USD by 2045 [1]. Similarly, diabetes has become an urgent health issue for China since 1980 [2, 3].

Incorporating glucose-lowering drugs (GLDs) into NRDL through negotiation which is a major innovation in China's reimbursement drugs list adjustment in recent years can significantly improves the availability and affordability of GLDs [4]. The economic evaluations (EEs) can significantly support decision-making of price negotiations for coverage and reimbursement of drugs included in the reimbursement list.

With Chinese health expenditure rapidly climbing, how to guarantee the continuous development of medical healthcare insurance fund becomes a hot issue. As an important tool to evaluate value of health technology, EEs have become a necessity, and more attention was paid on EEs studies in the context of reimbursement drugs list adjustment in China.

Decision-makers require clear interpretation of EEs results to inform reimbursement decisions [5]. Notably, the adequacy of reporting various elements of any health economic evaluation is imperative to benefit the understanding and interpretation of these studies. Additionally, transparency of reporting is an essential factor needed to evaluate EEs results [6]. Moreover, these published economic evaluations can partly reflect the economic evaluation evidence provided by manufacturers in drug price negotiation. Totally, there is a need to assess reporting quality of these EEs with EEs of targeting negotiated GLDs increasing.

Worldwide, the quality evaluation studies of EEs are limited, primarily focusing on different health services, including various therapies of main cancers causing much death [6–9], intervention for oral health [10, 11], traditional Chinese medicine [12] and other interventions. Also, various tools are used to assess the quality, such as Drummond's checklist, Consolidated Health Economic Evaluation Reporting Standards statement (CHEERS) checklist, Quality of Health Economic Studies (QHES) checklist, Grading of Recommendations Assessment, Development and Evaluation (GRADE) checklist and Philips' checklist. While the focus attention and evaluation instruments are diverse, the purpose of most studies are similarly regarded to support decision-making.

However, there has no study assessing the reporting quality targeting on negotiated GLDs in NRDL. Consequently, it is difficult for decision maker to understand and interpret the results of these studies because of the lack of the adequacy and transparency of reporting. To guide and further standardize the reporting of economic evaluations, the International Society for Pharmacoeconomics and Outcomes Research (ISPOR) issued the Consolidated Health Economic Evaluation Reporting Standards statement (CHEERS) [13]. The objective of the present study was to systematically identify and review published EEs related to GLDs included in the China NRDL (2020 version) and to assess their quality using CHEERS checklist as a reference.

## Materials and methods

### Literature sources and selection

A literature search was conducted through PubMed, Web of science, Embase (OVID), and Chinese databases (CNKI, Wan Fang Database, SinoMed, VIP) to identify economic evaluations of 12 negotiated GLDs in China NRDL (2020 version). Among 12 negotiated GLDs in NRDL (2020 version), Insulin and its analogues includes IDegAsp /Insulin Degludec/Insulin Aspart, α-glucosidase Inhibitors (AGI) includes acarbose, glucagon like peptide-1(GLP-1) and its analogues include exenatide, liraglutide, lixisenatide, benaglutide, dulaglutide, and polyethylene glycol loxenatide. Sodium-dependent glucose transporters 2(SGLT-2) includes dapagliflozin, empagliflozin, canagliflozin and ertugliflozin. The keywords included “cost effectiveness”, “cost minimization “, “cost utility”, “cost benefit”, “least-cost approach “, “pharmacoeconomics”, “economics”, “economic evaluation” and so on. Since the NRDL involved in the study comes into effect for use in March 2021, the search time was set up to March 2021. The search strategy is showed in Supplement 1. The searcher was restricted to publications written in English or Chinese due to the linguistic capabilities of the authors of this analysis.

Articles were included if they: 1) reported the economic evaluations; 2) were concerned about negotiated glucose-lowering drugs in NRDL;3) were set in Chinese mainland; 4) were written in English or Chinese.

Articles were excluded if they: 1) repeatedly reported economic evaluations; 2) were not concerned about negotiated glucose-lowering drugs in NRDL; 3) were set in countries other than Chinese mainland; 4) were systematic reviews, editorials, comments, or letters to the editor.

Based on the CHEERS checklist, the following data was identified and extracted from each selected EE into a Microsoft Excel spreadsheet: year of publication, journal type, main location of the first author and corresponding author, sponsor, methods of treatment, type of EEs (including cost effectiveness analysis, cost minimization analysis, cost utility analysis, cost benefit analysis), interventions and control, populations analyzed, study perspective, time horizon, whether the author's affiliations include the company to which the product belongs, study conclusion, choice of model and discount rate.

### Evaluation of studies

To provide recommendations, in the form of a checklist, to optimize reporting of health economic evaluations, the Consolidated Health Economic Evaluation Reporting Standards (CHEERS) statement [13] was produced by the CHEERS group and updated continuously [14]. Compared with other tools, it was intended to help authors provide accurate information on which health interventions are being compared and in what context, how the evaluation was undertaken, what the findings are, and other details that may aid readers and reviewers in interpretation and use of the study [13]. And it is one of the most used evaluation tools for quality evaluation of health economics. All recommendations of CHEERS are subdivided into six main categories: 1) title and abstract, 2) introduction, 3) methods, 4) results, 5) discussion, and 6) other [13]. The recommendations are contained in the CHEERS statement with 24-item checklist.

For each EE, items were scored as “fully met”, “not meet”, “partially met”, or “not applicable”. Since each item focuses on one single aspect, equal weights were allocated. Studies that fully met each of the items of the checklist were scored as ‘1’, items that partially met the criteria 0.5 and 0 when the study did not meet the criteria. We then calculated the score rate through the following formula: score rate = quality evaluation scores /full scores with applicable items *100%.

The quality assessment of included studies were performed by two reviewers independently (LL and BSY), in accordance with the CHEERS checklist [13] which provides a framework for assessing the reporting quality and the results of EEs study (Supplementary 2). The results were proofread, and any areas of disagreement were evaluated by the third reviewer (LFM) to reach a consensus. Ethical approval was not required for this study.

### Statistical analyses

Descriptive statistics summarizing the characteristics of the included EEs, the condition of CHEERS items and the results of CHEERS scores were reported by Microsoft Excel 2020. The Kruskal–Wallis test and Mann–Whitney U test were performed to explore the potential relationship between reporting quality and various characteristics of EEs using RStudio 2021.09.0. A *p*-value of < 0.05 was considered as the threshold for statistical significance.

## Results

### Literature search

**Fig. 1:**
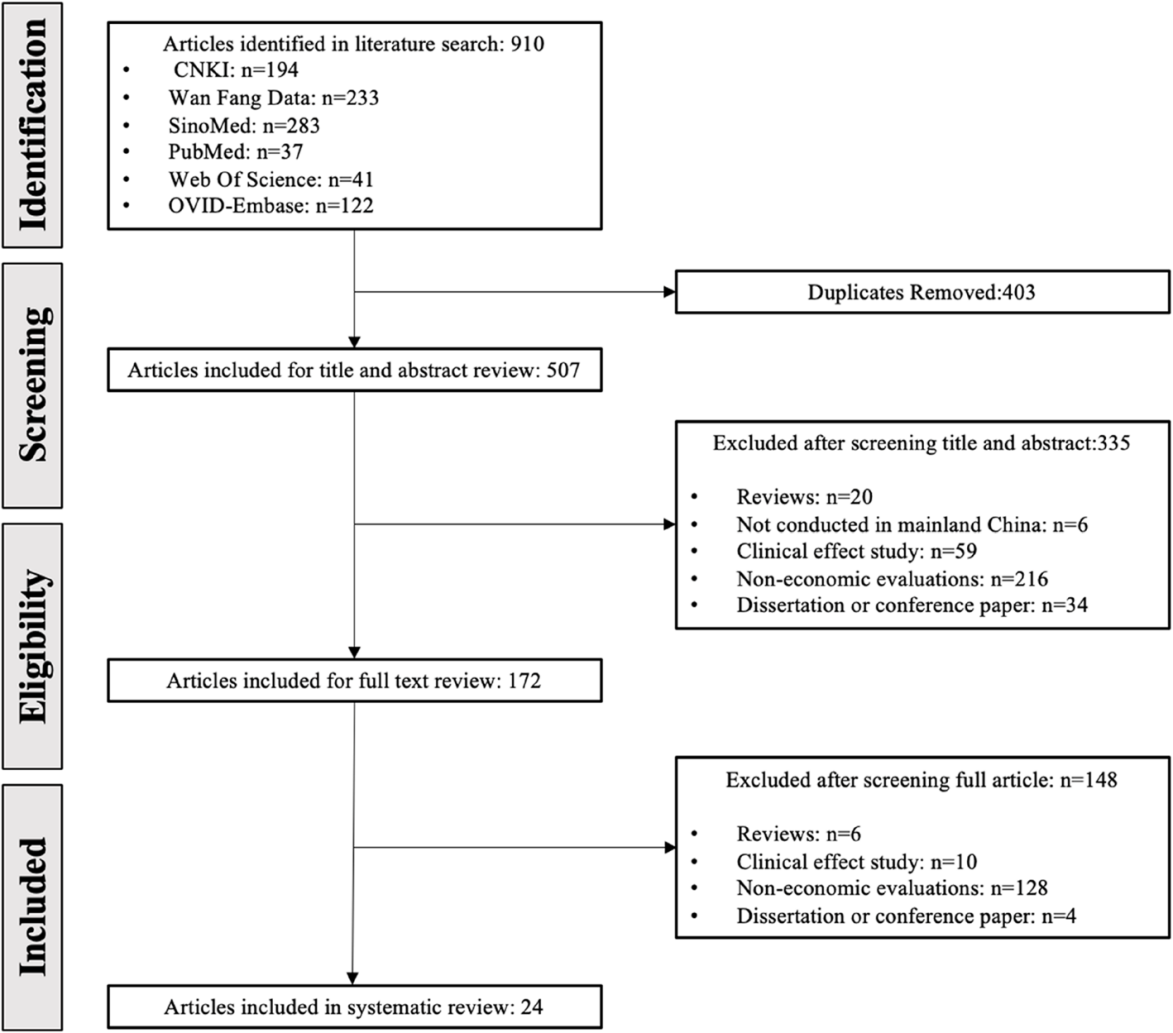
PRISMA 2020 flow diagram of literature review and screening

The systematic literature search conducted on CNKI, Wan Fang Database, VIP, Sinomed, PubMed, Web of science and Embase (OVID) identified 910 studies, among which 403 were duplicated and 335 EEs were excluded after screening the title, abstract that did not match the eligibility criteria (Fig. 1). A total of 172 EEs were included for full text review, among which 148 were excluded for several reasons: they were not EEs (*n* = 128); they were systematic reviews, conference abstracts, appraisals, or guidelines (*n* = 10); they were concerned about clinical effect instead of the economic evaluations (*n* = 59). Finally, 24 EEs were eligible for the present systematic review [15–38].

### Characteristics of the economic evaluations

The characteristics of the 24 EEs are presented in Table 1. EEs were first published in 2012, and there has been a considerable increase in published EEs ever since 2016. In generally, 14 EEs are published in English Journal, 10 are published in Chinese journal. All conclusions of EEs were made as whether they had economic value. First author of EEs mainly came from “hospital and university” in 33.33% of cases. Totally, 54.17% of cases were funded by government, companies, and other sponsors. The most common type of treatment was combination (*n* = 13, 54.17%).

**Table 1:** Basic characteristics of twenty-four included economic evaluations

	Number	Percentage
Selected EEs	24	100
Year of publication		
2012 ~ 2015	4	16.67
2016 ~ 2021	20	83.33
Journal type		
Chinese	10	41.67
English	14	58.33
First author affiliations		
research group	3	12.50
hospital	3	12.50
university	6	25.00
research group + university	3	12.50
hospital + university	8	33.33
research group + hospital + university	1	4.17
Sponsor		
No	11	45.83
Yes	13	54.17
Methods of treatment		
Independent	11	45.83
Combination	13	54.17
EEs type		
CEA	2	8.33
CUA	2	8.33
CBA	1	4.17
CMA	1	4.17
CEA + CUA	16	66.67
CEA + CMA	1	4.17
CEA + CUA + CBA	1	4.17
Study perspective		
healthcare system	9	37.50
societal	6	25.00
healthcare payers	2	8.33
Chinese healthcare. service providers	4	16.67
NR	3	12.50
Time horizon		
10 years	1	4.17
20 years	1	4.17
30 years	6	25.00
40 years	5	20.83
50 years	1	4.17
lifetime	6	25.00
NR	4	16.67
Discounted Rate		
3%	10	41.67
5%	9	37.50
3.5%	1	4.17
NR	4	16.67
Model Choice		
CORE	6	25.00%
Markov	3	12.50%
Cardiff	8	33.33%
others	3	12.50%
NA	4	16.67%

*NR* Not Report, *NA* Not Applicable

Generally, EEs included 4 types, including cost effectiveness analysis (CEA), cost utility analysis (CUA), cost benefit analysis (CBA) and cost minimization analysis (CMA). Two or more evaluation methods can be used simultaneously in a study, with one technique being used as the primary evaluation method and the other as a complement. Two thirds of the EEs are cost effectiveness analysis and cost utility analyses(*n* = 16). In three out of eight cases, authors conducted the analysis from the perspective of the health care system (*n* = 9). The time horizon of models ranged from 10 years to lifetime, with 25% (*n* = 6) of EEs with a “lifetime” perspective, 25% (*n* = 6) of EEs with a “30 years” perspective and only four EEs did not report it. Authors of EEs used a model in more than 83.33% of EEs (*n* = 18). Among 20 model-based EEs, Cardiff diabetes model (40%), IQVIA CORE diabetes model (30%) and Markov model (15%) were primarily adopted. A discounting of costs and/or effectiveness was made in more than 83.33% of cases (*n* = 18). Completed information extracted of the 24 selected EEs are presented in Supplementary 3.

### CHEERS scores of economic evaluations

**Fig. 2:**
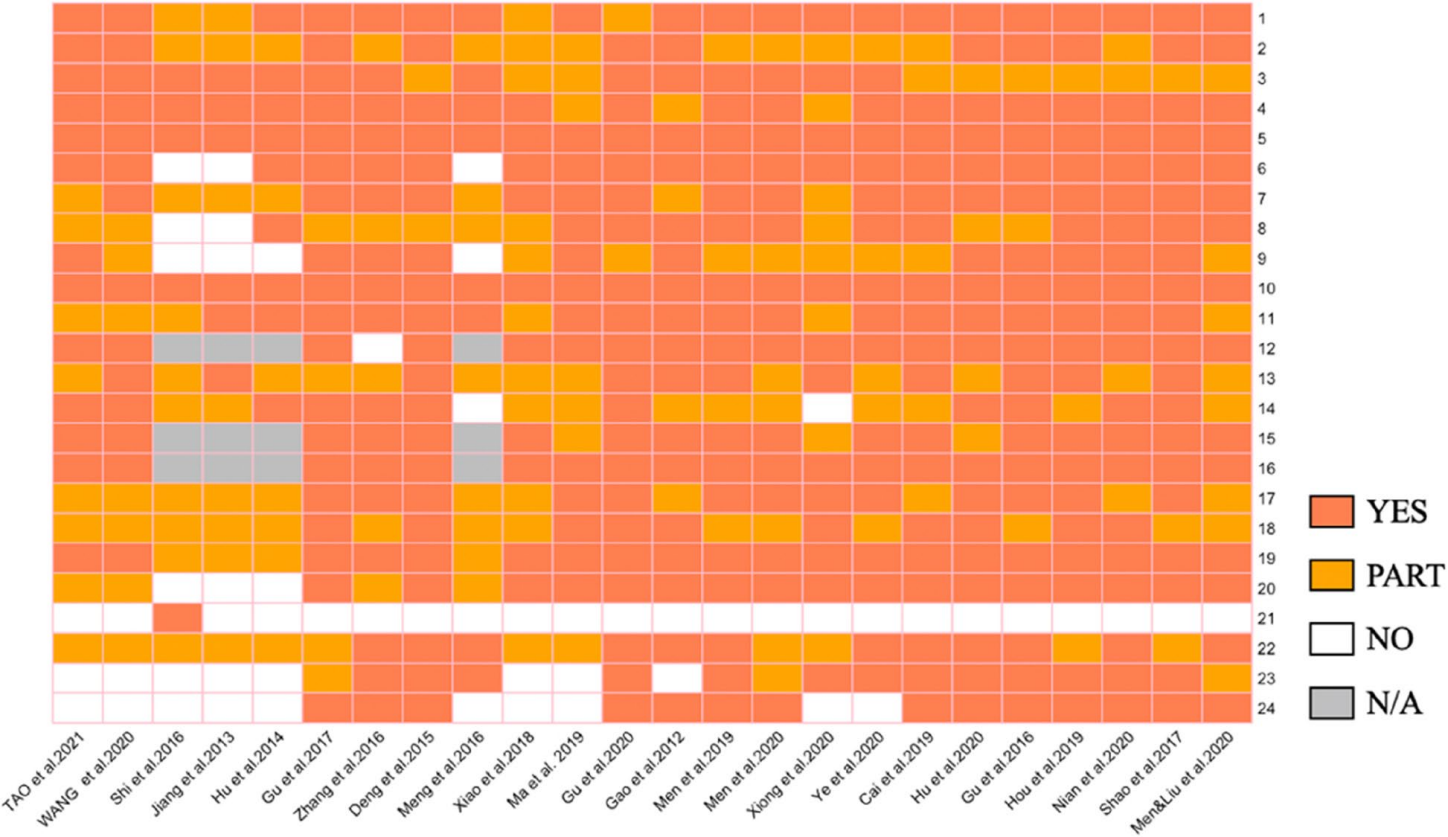
The reporting results of each appraised article based on the CHEERS checklist items

**Fig. 3:**
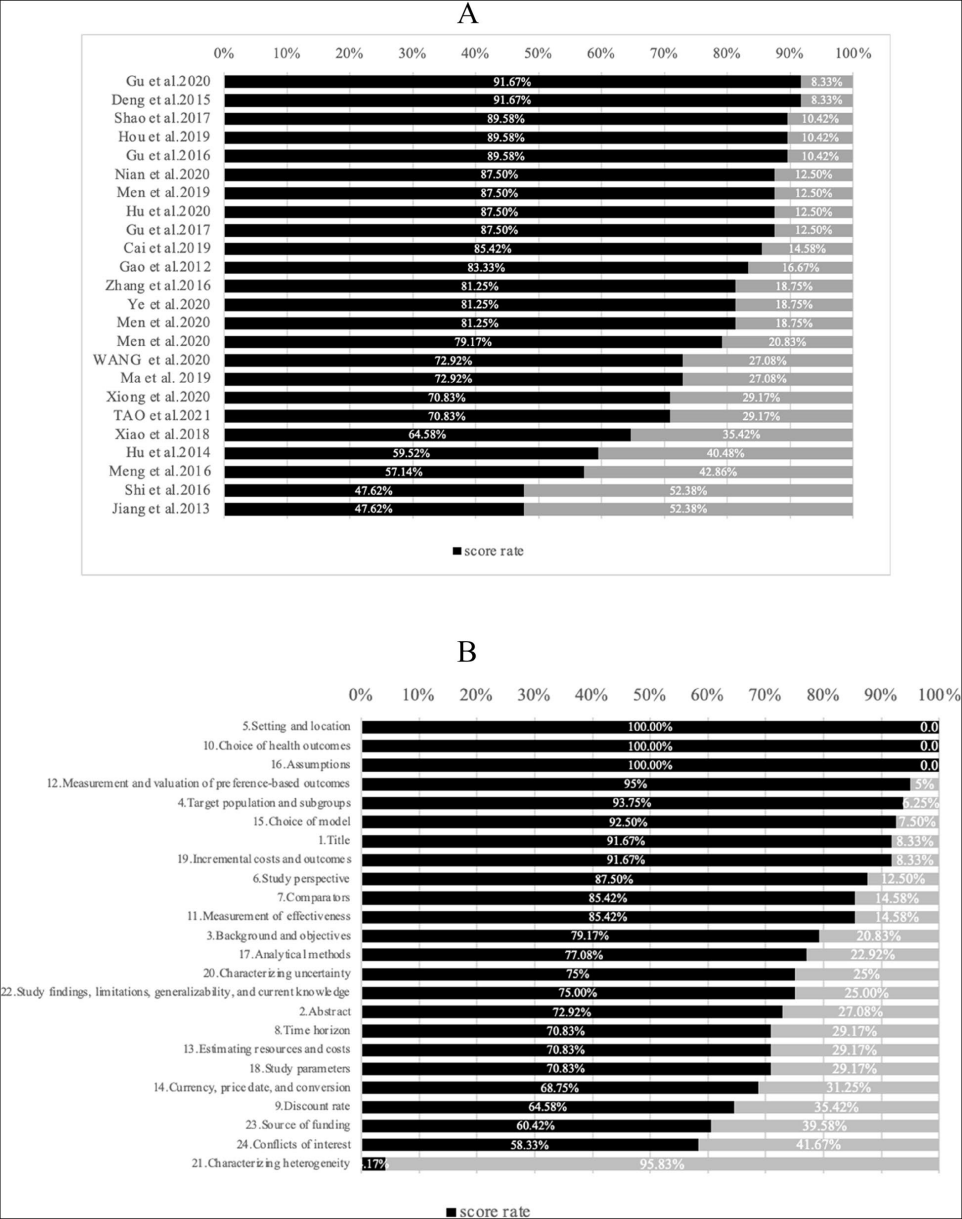
Overview of evaluation using CHEERS criteria, per article and per item. **A** Ranking of completeness of the 24-item CHEERS evaluation applied on the 24 selected studies. **B** Ranking of completeness of subitems

Based Figs. 2 and 3, the overall reporting quality score (with a maximum of 24) ranged from 10 to 22 (mean 18.31; SD 3.67; median 19.5). Scores for each article are illustrated in percentages for direct comparison. Similarly, the overall average score rate was 77.41 ± 13.23%. Two (8.33%) study [22, 26] had a score of 22 (91.67% of the items were scored positively). Two (8.33%) study [17, 18] had a score of 10 (47.62% of the items were scored negatively). Criteria that were often adequately described in the studies were the setting and location (score rate = 100%) and choice of health outcomes (score rate = 100%). Criteria that were least appropriately described were the characterizing heterogeneity (score rate = 4.17%). Completed scoring results of the 24 selected EEs based on CHEERS are presented in Supplementary 4.

**Fig. 4:**
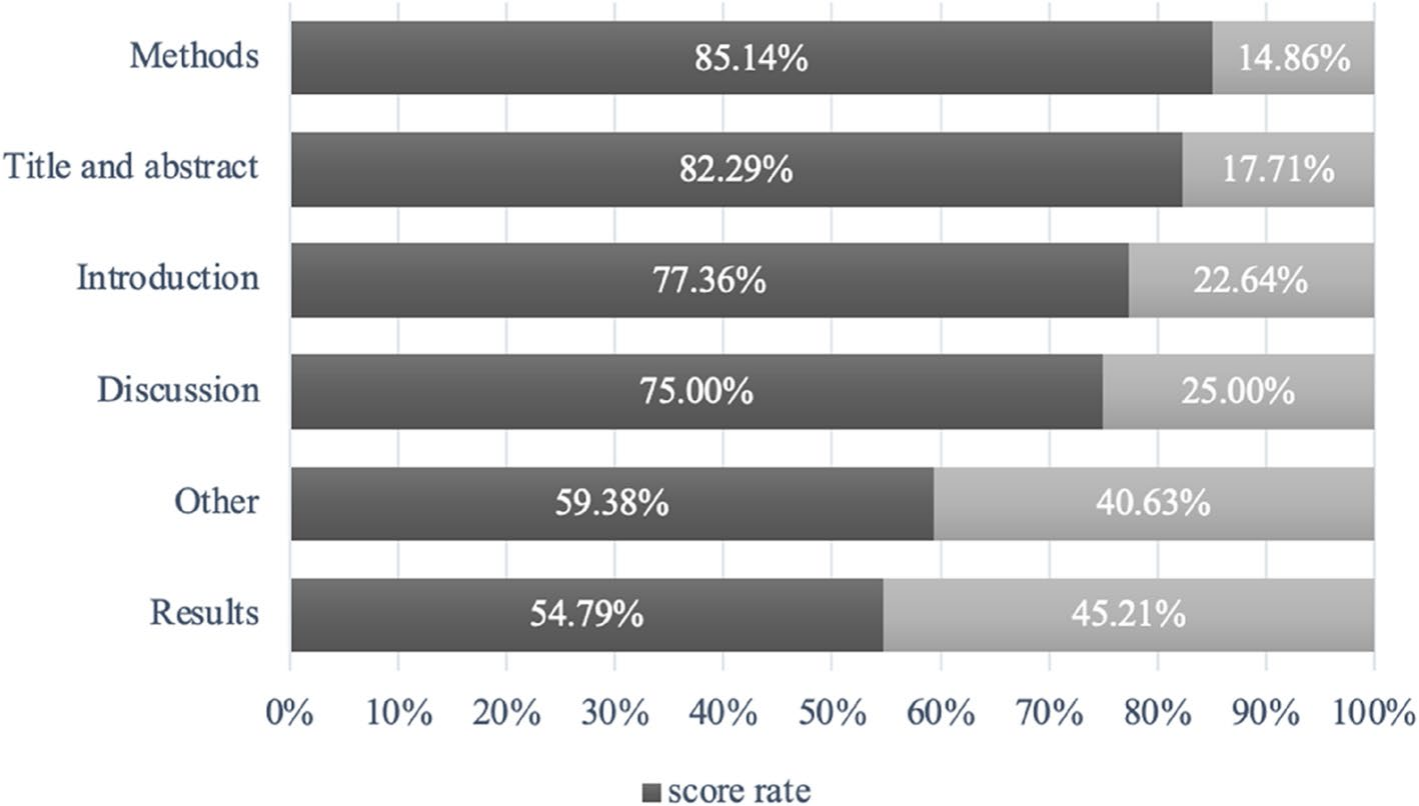
The proportion of studies that satisfy each major parts are detailed according to overall CHEERS instrument

The items of CHEERS were divided into six sections: title and abstract, introduction, methods, results, discussion and other. The score rate of each section was calculated as shown in Fig. 4. Methods section had the highest score rate of 85% while results section had the lowest score rate of 55%. Results section was consisted of four items including “Study parameters”, “Incremental costs and outcomes”, “Characterizing uncertainty” and “Characterizing heterogeneity”. The Incompleteness of “Characterizing uncertainty” mainly resulted in the lowest score rate of results section. There were four [17–19, 23] individual study-based economic evaluations, three [17, 19, 23] of which did not describe the impact of sample uncertainty on the estimation of incremental costs, incremental effects. In addition, only one [17] economic evaluation reported the effect of sample uncertainty on the estimated incremental costs, incremental effectiveness and incremental cost effectiveness but not the effect of the discount rate on uncertainty. Differences in costs, outcomes or ICERs may resulted from the populations with different baseline characteristics or different subgroups of intervention effects, which was missing from the other 23 economic evaluations.

### Quality scoring influencing factors

Based on extracted information, several shared characteristics of EEs included Journal Type, Published Year, EEs Type, Model Choice, Funding and Study perspective. Journal type depends on that identified EEs were published on Chinese journal or English journal. Published year shows the year of EEs publishment. The published years of EEs were divided into 2012 ~ 2015 and 2016 ~ 2021 because the Chinese first pricing negotiation happened in 2016. EEs Type contain 7 categories including CEA Separately, CUA Separately, CBA Separately, CMA Separately, CEA combined with CUA, CEA combined with CMA and CEA combined with CUA and CBA. Since the excessive classifications and the small sample size of each classification, EEs type was divided into CUA-related and CUA-unrelated to facilitate the analysis. Model Choice describes different analysis model in EEs studies. Funding means whether the identified EEs were funded by government, companies, or other sponsors. Study perspective includes perspectives of healthcare system, society, healthcare payers, Chinese healthcare service providers and not reported. The normality of the score rate was tested as Skewed distribution by s-w teat (W = 0.86521, *p*-value = 0.004245).Based on Mann–Whitney U Test Results (Table 2) and Kruskal–Wallis Test Results (Table 3), score rate which represented reporting quality of EEs was significantly related to journal type, EEs type, model choice and study perspective. Whether EEs type was CUA-related or CUA-unrelated, Mann–Whitney U Test Results for Published Year under different EEs Type (Table 4) showed that there was no significant difference in score rate between different Published years. It was revealed that HTA introduction had no statistically significant impact in score rate.

**Table 2:** Mann–Whitney U test results

Factors	Classification	Mean	SD	W	*P*-value
Journal Type	Chinese journal	64.52%	0.11	2	7.144 e^−05a^
	English journal	86.61%	0.04		
Published Year	2012 ~ 2015	70.54%	0.18	33	0.6129
	2016 ~ 2021	78.78%	0.12		
EEs Type	CUA-related	82.35%	0.08	7	0.004277^a^
	CUA-unrelated	58.63%	0.14		
Funding	Yes	81.96%	0.09	51.5	0.2563
	No	72.02%	0.16		

*NR* Not Report, *NA* Not applicable

F^1^: “^a^” means *P* < 0.05

**Table 3:** Kruskal–Wallis test results

Factors	Classification	Mean	SD	Chi-squared	*P*-value
Model Choice	CORE	76.39%	0.08	17.338	0.001662^a^
	Markov	75.00%	0.06		
	CDM	88.28%	0.03		
	Others	85.42%	0.06		
	NA	52.98%	0.06		
Study perspective	healthcare system	78.01%	0.09	11.23	0.0241^a^
	societal	79.37%	0.12		
	healthcare payers	86.46%	0.01		
	Chinese healthcare service providers	88.54%	0.01		
	NR	50.79%	0.05		

*NR* Not Report, *NA* Not Applicable

F^1^: “^a^” means *P* < 0.05

**Table 4:** Mann–Whitney U test results for published year under different EEs type

EEs Type	Published Year	Mean	SD	W	*P*-value
CUA-related	2012 ~ 2015	87.50%	0.06	9.5	0.3486
	2016 ~ 2021	81.74%	0.08		
CUA-unrelated	2012 ~ 2015	62.00%	0.17	2.5	1
	2016 ~ 2021	53.57%	0.08		

## Discussion

Economic evaluation is a necessary material for medical insurance price negotiation in China, and the quality of its report will affect the scientificity and reliability of decision-making. To benefit the understanding and interpretation of these studies, the adequacy of reporting various elements of any health economic evaluation is imperative. In addition, the economic evaluation materials of China's health care negotiations are not publicly available. This study reviews the published EEs of negotiated GLDs, and evaluates their quality according to CHEERS, to provide reference for future price negotiations of GLDs.

Our study included 24 published EEs about GLDs in NRDL and evaluated the reporting quality based on modified CHEERS, a reliable and valid measurement tool consisting of 24 items. The CHEERS items were adapted to the Chinese friendly version transferred from standard version in English, not only to clarify the meaning of the CHEERS entry, but also to make it easier for evaluators to understand and implement by refining the rules of the items in detail.

Due to some CHEERS items may be not applicable, it was difficult to compare exactly with each other by CHEERS evaluation scores of identified EEs. Converting scores into rates could be more comparable when comparing the reporting quality of different EEs or comparing the scores of different CHEERS items, making the reporting quality comparison more convincing. According to the previously reported by Rezapour et al. [39], quality scoring ≥ 85% were categorized as having excellent reporting quality, 70–85% as very good reporting quality, 55–70% as moderate reporting quality, and quality scoring < 55% were classified as poor reporting quality. The average score rate of all selected EEs in this study was 77.41%, which was identified as good reporting quality. Nevertheless, there remains room for improvement in the economic evaluations of negotiated GLDs in NRDL.

From the overview of quality evaluation using CHEERS criteria per article, the completeness of the EEs reporting varied between the different characteristics of the included studies, with quality ranging from low (47.62%) to high (91.67%).According to the score rate of different CHEERS items, the poor quality of reporting was mainly due to characterizing heterogeneity, with only 1 study out of 24 EEs reporting differences in costs, outcomes interpreted as differences between subgroups of patients with different baseline characteristics or other observed changes in effect.

Furthermore, any potential for conflict of interest of study contributors in accordance with journal policy and whether the study was funded and the role of the funder in the identification, design, conduct, and reporting of the analysis should be described adequately. Detailed information on potential conflict of interest (COI) and sponsorship was pivotal for the adequate understanding and appropriate interpretation of the reported study results [40]. The choice of discount rates used for costs and outcomes was reported in all model-based EEs, but the reasons of the choice were insufficient. The dates of the estimated resource quantities and unit costs was reported but the methods for adjusting estimated unit costs to the year of reported costs and for converting costs into a common currency base and the exchange rate should be reported clearly in EEs studies.

From differences of score rates between sections, results section qualified poorest among sections. Although the results section contained only two items, the items in the results section had more content to report compared to the other sections, and the corresponding items score points became less weighted, which leaded to a certain bias in the evaluation results [6]. At present, other quality assessment criterions have been developed internationally, including the Quality of Health Economic Studies (QHES) and BMJ checklist, etc. More applicable criterions for quality assessment studies could be chosen according to our needs, and we can also standardize the studies with these criteria, to improve the reporting quality.

Based on Kruskal Wallis test and Mann–Whitney U test results, journal type, EEs type, study perspective and model choice were linked to the quality of EEs reporting. The reporting quality of EEs published in English journals was higher than that of EEs published in Chinese journals, which may be partly due to the higher promotion of CHEERS abroad, most researchers in China still did not refer to the CHEERS standard, and Chinese journals did not have clear requirements for the completeness of content reporting of EEs. To further improve the reporting quality of EEs studies, these pharmacoeconomic evaluation criterions can be referred by Chinese journals for review process. The study found that CUA-related EEs were reported to be of better quality than CUA-unrelated EEs, which supported that CUA is the type of economic evaluation mostly recommended by China Guidelines for Pharmacoeconomic Evaluations (2020) [41]. The clarity of the study perspective in EEs was closely related to the cost components and efficacy determination, which varied under different study perspectives and outcome indicators and may affect the quality of the reporting. The quality of reporting varied with the model chosen, but the sample size is limited that more studies should be conducted to support the conclusions of influencing factors.

To our knowledge, quality evaluation of EEs for negotiated GLDs has not been studied before. Quality evaluations could identify high qualified EEs and provide evidence for NRDL scientific adaptation decision reference. The CHEERS checklist is gradually recognized and used in quality assessment areas with time going. In China, some economic evaluation scales like CHEERS should be promoted for quality evaluation as one of the important tools. However, there are some limitations in our study. There is no breakdown of the weighting of each score point for each item. In other words, the scoring rules for this study do not reflect the extent to which the reported economic evaluations meet CHEERS checklist. Besides, with only 24 economic evaluations included in the study, the sample size may cause uncertainty to explain the results of Kruskal-Wallis tests. CHEERS checklist is only applicable to the evaluation of the reporting quality of economic evaluations and unable to study the quality of the economics evaluation process [42]. As CHEERS 2022 had not been published at the time of completion of this study, the version of CHEERS used in this study was CHEERS 2013.

## Conclusion

We provide a comprehensive summary and a systematic review of the EEs quality evaluation about negotiated GLDs in NRDL, finding that reporting quality was good according to our results. However, there remain rooms for reporting quality to be substantially improved in some aspects including characterizing heterogeneity, potential conflict of interest (COI) and sponsorship and others. Findings here clearly demonstrate many scales like CHEERS could not only work as an internationally accepted standardization checklist but also a quality evaluation tool. Additionally, because economic materials need to be addressed and reported in a drug reimbursement application, both pharmaceutical companies and the government could report relevant EEs results according to the standard checklist to get qualified reports for reference and cooperate hormonally with each other.

### Supplementary Information

**Supplementary Material 1.**

**Supplementary Material 2.**

**Supplementary Material 3.**

**Supplementary Material 4.**

## Data Availability

All data generated or analyzed during this study are included in this published article and its supplementary information files.

## Abbreviations

GLDs: Glucose-lowering drugs
NRDL: National Reimbursement Drug List
CHEERS 2013: Consolidated Health Economic Evaluation Reporting Standards 2013
USD: US dollars
EEs: Economic evaluations
QHES: Quality of Health Economic Studies
GRADE: Grading of Recommendations Assessment, Development and Evaluation
ISPOR: International Society for Pharmacoeconomics and Outcomes Research
AGI: α-Glucosidase Inhibitors
GLP-1: Glucagon like peptide-1
SGLT-2: Sodium-dependent glucose transporters 2
CEA: Cost effectiveness analysis
CUA: Cost utility analysis
CBA: Cost benefit analysis
CMA: Cost minimization analysis
COI: Conflict of interest
