# Supplementary material for: A systematic review on reporting quality of economic evaluations for negotiated glucose-lowering drugs in China national reimbursement drug list
Source: BMC Health Serv Res. 2024 May 1;24:562. doi: 10.1186/s12913-024-11001-3 (PMC11064232; doi:10.1186/s12913-024-11001-3)
Supplement: Supplementary file 1 — Supplementary Material 1. [file 12913_2024_11001_MOESM1_ESM.docx]

**Search Strategy**

**Take exenatide as an example**

**PubMed:**

1. "health economic"[Title/Abstract] OR "economic"[Title/Abstract] OR "pharmacoeconomic"[Title/Abstract]
2. "cost benefit analysis"[MeSH Terms] OR ("cost benefit"[All Fields] AND "analysis"[All Fields]) OR "cost benefit analysis"[All Fields] OR ("cost"[All Fields] AND "benefit"[All Fields] AND "analysis"[All Fields]) OR "cost benefit analysis"[All Fields] OR ("cost effectiveness analysis"[MeSH Terms] OR ("cost effectiveness "[All Fields] AND "analysis"[All Fields]) OR "cost effectiveness analysis"[All Fields] OR ("cost"[All Fields] AND "effectiveness"[All Fields] AND "analysis"[All Fields]) OR ("cost utility analysis"[MeSH Terms] ) OR ("cost utility"[All Fields] AND "analysis"[All Fields]) OR ("cost"[All Fields] AND "utility"[All Fields] AND "analysis"[All Fields]) OR "cost utility analysis"[All Fields]) OR ("costs and cost analysis"[MeSH Terms] ) OR ("costs and cost"[All Fields] AND "analysis"[All Fields]) OR ("costs"[All Fields] AND "cost"[All Fields] AND "analysis"[All Fields]) OR "costs and cost analysis"[All Fields] OR ("cost minimization analysis"[MeSH Terms]) OR "cost minimization” [All Fields] AND “analysis"[All Fields]) OR ("cost"[All Fields] AND "minimization"[All Fields] AND "analysis"[All Fields]) OR "cost minimization analysis"[All Fields])
3. "CBA"[All Fields] OR "CEA"[All Fields] OR "CUA"[All Fields] OR "CMA"[All Fields])
4. " exenatide"[Supplementary Concept] OR " exenatide "[All Fields]
5. "asian continental ancestry group"[MeSH Terms] OR ("asian"[All Fields] AND "continental"[All Fields] AND "ancestry"[All Fields] AND "group"[All Fields]) OR "asian continental ancestry group"[All Fields] OR "Chinese"[All Fields] OR ("China"[MeSH Terms] OR "China"[All Fields] OR "Chinas"[All Fields] OR "Chinas"[All Fields])
6. ① OR ② OR ③
7. ④ AND ⑤ AND ⑥

**web of science**

TS=(cost benefit analysis) OR TS=(cost effectiveness analysis) OR TS=(cost utility analysis) OR TS=(cost minimization analysis) OR TS=(health economic) OR TS=(economic) OR TS=(pharmacoeconomic)

1. TS=(CMA) OR TS=(CEA) OR TS=(CUA) OR TS=(CBA)
2. ① OR ②
3. TS=(Chinese) OR TS=(China)
4. TS=(exenatide)
5. ③ AND ④ AND ⑤

**OVID (Embase):**

1. exp economics/ OR exp "costs and cost Analysis"/ OR exp cost benefit analysis/ OR (“cost benefit analysis” OR “cost utility analysis” OR “cost effectiveness analysis” OR “cost minimization analysis” OR “cost benefit” OR “cost effectiveness” OR ” cost utility” OR ” cost minimization” OR "costs and benefits" OR economic evaluation marginal analysis).mp.
2. asian continental ancestry group/ OR chinese.mp. OR china/ OR (china OR mainland china OR "people's republic of china").mp.
3. exenatide.mp.
4. ① AND ② AND ③

**CNKI**

SU=( cost benefit + cost effectiveness + cost utility + cost minimization + benefit cost + benefit evaluation + economic benefit + minimum cost + pharmacoeconomic + economic)* Exenatide

**Wan Fang Data**

Subject: (" cost effectiveness "OR" cost utility "OR" cost benefit "OR" cost minimization "OR" least-cost "OR" pharmacoeconomic" OR "economic" OR "economic evaluation") AND (" exenatide ")

**Sinomed**

1 cost effectiveness OR cost benefit OR cost minimization OR cost utility OR least-cost method OR pharmacoeconomic OR economic OR economic evaluation

2 (" cost benefit analysis/economics "[unweighted: Extension]) OR "costs and cost analysis/economics "[unweighted: extension]

3 (#2) OR (#1)

4 exenatide

5 (#4) AND (#3)
